# Probiotic of *Lactiplantibacillus plantarum* NWAFU-BIO-BS29 Isolated from Chinese Traditional Fermented Milk and Its Potential Therapeutic Applications Based on Gut Microbiota Regulation

**DOI:** 10.3390/foods11233766

**Published:** 2022-11-23

**Authors:** Mohamedelfatieh Ismael, Yaxin Gu, Yanlong Cui, Tao Wang, Fangfang Yue, Yanting Qin, Xin Lü

**Affiliations:** 1 College of Food Science and Engineering, Northwest A&F University, Yangling, Xianyang 712100, China; 2 Sudanese Standards and Metrology Organization, Khartoum 13573, Sudan; 3 College of Food Science, China Agricultural University, Beijing 100083, China

**Keywords:** probiotic, *Lactiplantibacillus plantarum*, virulence genes, antioxidants, hemolytic activity, cholesterol-lowering, gut microbiota

## Abstract

Lactic acid bacteria are one of the bioresources that can promote the host’s health and have potential therapeutic applications. This study aimed to evaluate the probiotic properties of novel *Lactiplantibacillus plantarum* NWAFU-BISO-BS29 isolated in vitro from traditional Chinese fermented milk, assess its safety, and study its interaction with the gut microbiota using a BALB/c mouse model. The findings reveal that this strain had a high tolerance to gastric acidity (64.4%) and bile salts (19.83–87.92%) with remarkable auto-aggregation and co-aggregation abilities (33.01–83.96%), respectively. Furthermore, it lowered the cholesterol levels in dead cells (44.02%) and live cells (34.95%) and produced short-chain fatty acids (SCFAs). Likewise, it showed good antioxidant properties and strong antipathogen activity against *Escherichia coli* and *Staphylococcus aureus* with inhibition zones at 21 and 25 mm, respectively. The safety assessment results indicate that all of the virulence factor genes were not detected in the whole DNA; additionally, no hemolysis or resistance to antibiotics commonly used in food and feed was observed. Interestingly, the 16S rRNA gene sequencing of the mouse gut microbiota showed a marked alteration in the microbial composition of the administrated group, with a noticeable increase in Firmicutes, Patescibacteria, Campylobacterota, Deferribacterota, Proteobacteria, and Cyanobacteria at the phylum level. The modulation of gut microbial diversity significantly improved the production of SCFCs due to the abundance of lactobacillus genera, which was consistent with the functional gene predictive analysis and is believed to have health-promoting properties. Based on these results, our novel strain is considered a safe and good probiotic and could hold high potential to be used as a starter culture or to safely supplement functional foods as a probiotic and may provide new insights into therapeutic interventions.

## 1. Introduction

The Food and Agriculture Organization (FAO) and World Health Organization (WHO) defined probiotics as live microorganisms that, when administered in appropriate amounts, provide a health benefit to the host [1,2]. In recent years, lactic acid bacteria (LAB) have attracted growing interest among consumers globally due to their positive role in promoting health, such as enhancing the immunity system, improving the balance of gut microflora, reducing the population of harmful microorganisms, increasing the tolerance to lactose-containing foods, and providing therapeutic properties, such as preventing cancer (antitumor activity), preventing some intestinal colitis and some pathogenic diseases (antimicrobial), and possibly lowering cholesterol [3,4]. The US Food and Drug Administration (FDA) recognized LAB as being safe to use as probiotics in food and feed [5,6]. The WHO report of 2015 mentioned that a 10% reduction in serum cholesterol could decrease the incidence of heart disease within five years by 50% in 40-year-old men [7,8]. Indeed, numerous attempts have been made to isolate cholesterol-reducing LAB to study the relationship between intestinal microbiota and cholesterol metabolism. However, the genes or enzymes involved in this metabolism are still unknown [9]. Gastrointestinal illnesses can cause considerable mortality as well as enormous economic loss. LAB have the ability to produce natural antimicrobial metabolic substances to kill or inhibit food-borne pathogens, such as organic acids, hydrogen peroxide, and bacteriocins, which represents a promising contribution to food safety as bio-preservatives [10,11].

The criteria used to mine putative probiotic strains for human and animal administration consist of evaluating their desirable properties, such as bacterial adhesion ability to colonize the intestinal area of the host via bacteria clumping in the form of auto-aggregation with the same strain or coaggregation with different strains to prevent pathogen colonization and survival under gastric gut conditions [12]. Additionally, antioxidant activity shows a potential function in reducing oxidative damage, which can cause degenerative diseases such as cancer, Alzheimer’s disease, and Parkinson’s disease, during cellular metabolism in the body by delaying or preventing the oxidation of the cellular substrates of reactive oxygen species (ROS), such as hydrogen peroxide (H_2_O_2_), superoxide anion (O_2_-), and hydroxyl radical (-OH), which play important roles in cell signaling, apoptosis, gene expression, and ion transportation [13]. In addition, there are safety assessments to ensure probiotic strains do not have antibiotic resistance, hemolysis activity, or virulence-carrying factor (VF) genes, which can potentially be transferred horizontally to the flora of humans, animals, or to the pathogenic bacteria temporarily residing in the hosts [14,15]. Furthermore, the production of short chain fatty acids (SCFAs), such as acetic acid, butyric acid, propionic acid, isobutyric acid, isovaleric acid, and valeric acid, by LAB has essential impacts on innate immunity, as they act as anti-inflammatory inhibitors and regulators of the gut microbiota [16].

The gut microbiota of humans comprises approximately 10^14^ microorganisms, including viruses and yeast, and is an integral part of the human body and bacteria, with the diversity of microbiota affecting environmental factors [17]. The microbiota’s interaction with the host promotes the intestinal epithelial barrier, improves immune homeostasis, protects from pathogen colonization, and inhibits deleterious inflammatory reactions. Therefore, the gut microbiota’s composition can affect the normal mucosal immune system, while the imbalances in the gut microbiota’s composition, termed dysbiosis, can trigger several immune disorders through the activity of the adaptive immune system [18].

Fermented dairy products are considered an interesting and rich bioresource for mining probiotic microorganisms with functional properties [19,20]. In 1974, Mann and Spoerry reported that LAB from dairy products was associated with lowering serum cholesterol in African Maasai warriors [21]. Many strains isolated from fermented food have essential biological functions with antioxidant activity, such as *Lactobacillus casei*, *Lactobacillus rhamnosus* GG, *Lactobacillus fermentum* ME-3, and *Lactobacillus brevis* BJ20 [22].

The application of specific Lactobacillus spp. strains is still limited because probiotics have species- and strain-specific effects and the mechanisms have not been fully elucidated. Accordingly, the present study aims to evaluate the potential probiotic features of *Lactiplantibacillus plantarum* NWAFU-BIO-BS29 isolated from Chinese traditional fermented cow milk [23]. In vitro and in vivo investigations were conducted to assess the safety of *Lactiplantibacillus plantarum* NWAFU-BIO-BS29 by testing its hemolysis activity and susceptibility against eight antibiotics and detecting 16 genes related to the VFs as well as their tolerance to acidic and bile salts, their cholesterol-lowering activity, their antibacterial activity, and their aggregation activity. In addition, antioxidant activity includes scavenging activity against ABTS and hydroxyl, superoxide anion, and 2,2-diphenyl-1 picrylhydrazyl (DPPH) free radicals, and its resistance to hydrogen peroxide, as well as the production of SCFAs and their underlying mechanisms to modulate gut microbiota; they will be evaluated by testing the 16S sequence of feces from mice model. According to the findings of this study, the novel strain NWAFU-BIO-BS29 could be used as a promising probiotic with potential properties to promote health benefits and produce bio-preservative compounds.

## 2. Materials and Methods

### 2.1. Isolation and Molecular Identification of Lactiplantibacillus plantarum NWAFU-BIO-BS29

*L. plantarum* NWAFU-BIO-BS29 was previously isolated in the laboratory of Food Bioresources at NWAFU from traditional Chinese fermented milk collected from Gansu province. China [23]. The strain was stored at −80 °C in MRS broth (LAND BRIDGE, China) with 40% (*v*/*v*) glycerol (KESHI Chemical, Beijing, China) until use. For all in vitro investigations, a bacterial concentration of 10^8^ CFU/mL was used.

The genomic DNA of the potential probiotic strain was extracted by following the protocol of the purification kit (Sangon Biotech, Shanghai, China) [24]. Then, a polymerase chain reaction (PCR) for 16S rDNA amplification was carried out using a set of primers F27 and R1492. The PCR products were sequenced by Yangling Tianrun Aoke Biotechnology Co, Ltd. Sequencing department. Yangling, China, by means of SeqMan 12.2 software. The BLAST tool on the NCBI sequence database (https://blast.ncbi.nlm.nih.gov/Blast.cgi. accessed on 1 July 2022) was used to identify the obtained sequence at the level of species [25]. Molecular Evolutionary Genetics Analysis (MEGA) 11.0 software (https://www.megasoftware.net/. accessed on 1 July 2022) was used to construct the phylogenetic tree by using the neighbor-joining method [26].

### 2.2. Probiotics Properties

#### 2.2.1. Antibacterial Activity and Acid and Bile Salt Tolerance

The antibacterial activity was performed by using the agar well diffusion assay method (AWDA) as described by Sui and Liu [1] and Pinto and Barbosa [2] with some differences—in short, LAB was cultured in MRS broth (LAND BRIDGE, Beijing, China) at 37 °C overnight. Then, the cell-free supernatant (CFS) was collected by means of refrigerated centrifugation at 6000 rpm (HC-3016R, high-speed refrigerated centrifuge, Zonkia. Co., Ltd., Hefei, China). Then, 200 µL of CFS was poured into wells made by Oxford cups placed in double-layer Petri dishes of 1.5% agar overlaid with semi-solid LB medium (LAND BRIDGE, Beijing, China) (0.75% agar) containing 10^6^ cfu/mL of Gram (+ and −) indicators. The plates were put in a refrigerator at 4 °C to allow the diffusion of CFS for 1.5 h, and after that were incubated at 37 °C for 24 h, and subsequently we measured the diameter of the inhibition zones. *Escherichia coli* ATCC25922 and *Staphylococcus aureus* ATCC25923 were used as indicator strains.

As a simulation of the acidic environment of the human stomach, acid and bile salt tolerance was evaluated according to the methods of [3,5,24]. Briefly, the strain was cultured for 24 h, washed twice with sterile phosphate buffer saline (PBS. pH 7.0) (G-CLONE, China), and then re-suspended in MRS broth (LAND BRIDGE, Beijing, China). The pH was adjusted to 3.0 by HCl (XILONG SCIENTIFIC, China) and incubated at 37 °C for 3 h. The absorbance was measured at 600 nm after 0 and 3 h, respectively. The survival rate (SR) was calculated according to Equation (1):

(1) $$\mathrm{SR}\;\%=\;\left(A_{1}/A_{0}\right)\times 100$$

where A_1_ is the absorbance of LAB culture after treatment, and A_0_ is the absorbance before treatment.

The bile tolerance test was conducted in a similar manner as above. The strain was cultivated in MRS broth supplemented with 0.3%, 0.5% and 1.0% oxgall bile salt (MACKLIN, China), and then incubated at 37 °C for 12 h. The absorbance was measured at 600 nm [6]. SR was calculated according to Equation (1): A_1_ (absorbance of LAB culture), A_0_ (absorbance of the blank sample).

#### 2.2.2. Auto-Aggregation and Co-Aggregation Ability

Auto-aggregation activity was measured as described by [5,10,27] with some changes. An overnight culture was centrifuged at 8000 rpm for 10 min by using a high-speed refrigerated centrifuge, followed by washing twice and resuspending in PBS. Equal volumes of each suspension were mixed with broth and incubated at 37 °C for 14 h. The absorbance of cell suspensions was determined at 600 nm (UNICO7200 SPECTROPHOTOMETER, Shanghai Instrument, Shanghai, China). The auto-aggregation ability (AA) was calculated using Equation (2):

(2) $$\mathrm{AA}\;\%=\left(1-N_{\mathrm{time}}/N_{\mathrm{initial}}\right)\times 100$$

where A_initial_ and A_time_ are the absorbance before and after incubation, respectively.

A total of 1 mL of the cell suspension was mixed with an equal proportion of hydrocarbons (xylene).

The co-aggregation ability (CA) was assessed by resuspending 3 mL of LAB overnight culture and an equal volume/concentration of *E. coli* in PBS. Equation (3) was used to compute CA by measuring the absorbance of suspensions directly (A_m0_) and again after incubation for 4 h at 37 °C (A_m4_) [12].

(3) $$\mathrm{Aco}\;\%=\left(A_{m0}-A_{m4}\right){\;A}_{m0}\times 100$$

#### 2.2.3. Cholesterol-Lowering Ability

Cholesterol-lowering ability was assessed for live and dead cells of LAB in vitro as reported by [7,24,25,28] with some modifications. First, 2 mL of pre-activated culture was harvested using a high-speed refrigerated centrifuge from ZONKIA, followed by washing the pellets twice with PBS. For dead cells, the suspensions were autoclaved (BOXUN, Shanghai Boxun Industry & Commerce, Shanghai, China) at 121 °C for 15 min. Second, MRS broth (LAND BRIDGE, Beijing, China) containing 0.3% (*w*/*v*) oxgall bile (MACKLIN, Shanghai Macklin Biochemical, Shanghai, China) and 100 mg/L water-soluble cholesterol (SIGMA-Aldrich, Tokyo, Japan) was inoculated with 2% *v*/*v* of bacterial suspension of live and dead cells as mentioned above, and then incubated at 37 °C for 24 h. Nill MRS broth was used as a negative control. After that, the supernatant was collected by centrifugation of the suspension at 8000 rpm for 15 min. To measure the residual cholesterol, 1 mL of supernatant was mixed strongly with 1 mL of KOH (33% *w*/*v*) (Guangdong Guanghua Sci-Tech, Guangdong, China) and 2 mL of absolute ethanol (KESHI Chemical, Beijing, China), and this mixture was heated (KEWEI, Beijing, China) at 37 °C for 15 min then cooled to 25 °C. Subsequently, 5 mL hexane (GHTECH, Guangdong Guanghua Sci-Tech. Guangdong, China) and 2 mL distilled water (MOLECULAR H_2_O, Chongqing, China) were added to the mixture, which was then left at room temperature for 10 min to separate the hexane layer. The residues were immediately dissolved in 4 mL of o-phthalaldehyde (Aladdin, Shanghai, China) with 1 mL of sulfuric acid and then incubated for 10 min. The absorbance was measured at 560 nm for live and dead cells.

(4) $$\mathrm{CRR}\;\%=\left[100-\left({\mathrm{Cholesterol}}_{\mathrm{residual}}/{\mathrm{Cholesterol}}_{\mathrm{initial}}\right)\right]\times 100$$

where Cholesterol_residual_ and Cholesterol_intial_ are the absorbance of uninoculated and inoculated samples, respectively.

### 2.3. Assay of Safety Aspects

#### 2.3.1. Antibiotic Susceptibility Test and Hemolytic Activity

The antibiotic susceptibility of the LAB strain was assessed according to the Clinical and Laboratory Standards Institute Technical Guidelines (2017) as described by [10,27] with slight alterations by spreading 100 μL of 10^8^ CFU/mL bacterial culture on the surface of MRS agar containing antibiotics. The plates were incubated at 37 °C for 24 h to count the colonies. The following eight antibiotics were tested within the range of concentrations given in parentheses (mg/L): kanamycin (0.5–2.0), tetracycline (0.02), vancomycin (1.0) produced by (DIYIBIO, Shanghai, China), ampicillin (0.005), streptomycin (0.25–0.3), erythromycin (0.005), chloramphenicol (1.5–2.0), and gentamycin (0.25–0.3) produced by (MP Biomedicals, Strasburg, France). The concentrations outside the test range of the corresponding antimicrobial are available at JAC Online (http://jac.oxfordjournals.org/, accessed on 15 July 2022).

The hemolytic activity was evaluated according to the method described by Pieniz, de Moura [28]. Briefly, the strain was inoculated into blood agar (Qing Dao Hope Bio-Technology, Qingdao, China) supplemented with 5% *v*/*v* of defibrinated sheep blood (Beijing LandBridge Technology, Beijing, China) and then incubated at 37 °C for 72 h. The evaluation was performed by observing zones of hydrolysis around the colonies; strains that displayed a clear zone were considered as positive hemolytic (b-hemolysis), while the presence of a green-hued zone was considered to indicate partial hemolysis (a-hemolysis), while negative strains did not show any zones around the colonies (g-hemolysis).

#### 2.3.2. Detection of Virulence and Resistance Genes

The presence of 16 virulence factor genes (VFs) was detected according to the methods described by [29,30]. The VFs were related to: chemotactic factors (*cpd*), aggregation (*asa1*), hyaluronidase (*hyl*), Enterococcus factors (surface protein) (*mur-2ed*), genetic exchange (*Int*), gelatinase (*fsrA*), endocarditis (*efaA*), biogenic amine genes of histidine (*hdc1*), tyrosine (*tdc*), ornithine (*odc*), antibiotic resistance genes of tetracyclines (*tet(K)*), streptomycin (*ant(6)-Ia*), the aminoglycoside gentamicin (*aac(6′)-Ie-aph(2″)-Ia*), the glycopeptide vancomycin (*vanC2*), streptogramin (*vat(E)*), the macrolide erythromycin (*ermA*) and other chloramphenicols (*catA*). All of the primers were synthesized by the (Synthesis Laboratory, Yangling Tianrun Aoke Biotechnology, Yangling, China); sequences are listed in Table S1. The DNA was extracted from the potential probiotic using a kit from (Sangon Bio-tech, Shanghai, China) at a gDNA concentration of 100–200 ng/μL. Then, the polymerase chain reaction (PCR) was performed on BIO-RAD T100TM Thermal Cycler, Singapore, with a total volume of 25 μL (2 μL of forward and reverse primers, 2 μL gDNA, 12.5 μL 2 × Rapid Taq Green master Mix and 6.5 μL nuclease-free water) followed by loading the PCR products on 3.0% stained agarose gel by 0.005% (*w*/*v*) gel red (DIYIBIO, Shanghai, China), and subsequently, electrophoresis on the power supply (BIO-RAD PowerPacTM Basic, Singapore). The gel was visualized under ultraviolet light (BIO-RAD, Molecular Imager, Hercules, CA, USA).

### 2.4. Antioxidant Activity Analysis

Antioxidants are molecules that can receive electrons and/or donate hydrogen [31]. The antioxidant activity of the LAB culture extract was evaluated based on the reduction in free radicals of 2,2-diphenyl-1-picrylhydrazyl free radical (DPPH), 2,2′-azino-bis(3-ethylbenzothiazoline-6-sulfonate (ABTS), hydroxyl radical scavenging activity (HR), superoxide anion scavenging activity (SA) and resistance to hydrogen peroxide (RHP).

The assay of DPPH was conducted according to the photometric methods of Sui and Liu [1] and Li and Zhao [32] at an absorbance of 517 nm (UNICO7200 SPECTROPHOTOMETER, Shanghai Instrument, China). Equation (5) was used to calculate the DPPH percentage.

(5) $$\mathrm{SADPPH}\;\%=\left[\left(A_{\mathrm{sample}}-A_{\mathrm{blanck}}\right)/\left(A_{\mathrm{control}}-A_{\mathrm{blank}}\right)\right]\times 100$$

where A_sample_ is the absorbance of suspension, A_blank_ is the absorbance of only cells and ethanol, and A_control_ is the absorbance of the deionized water and DPPH solutions.

The ABTS test is an electron transfer-based assay. Wang and Shao’s [33] method was used to assess it. The readings were taken at an absorbance of 734 nm. Equation (6) was used to calculate the ABTS radical scavenging activity:

(6) $$\mathrm{SAABTS}\;\%=\left[1-\left(A_{x}-A_{b}/A_{0}\right)\right]\times 100$$

where A_x_ is the blank absorbance by replacing EPS solution with water, A_b_ is the sample solution, and A_0_ is the sample control.

The HR radical scavenging assay was determined using the sulfosalicylic acid method as described by Azat and Liu [5]. The absorbance was determined at 510 nm. Equation (7) was used to calculate the HR scavenging activity.

(7) $$\mathrm{SAHR}\;\%=\left[1-\left(A_{s}-A_{b}/A_{c}\right)\right]\times 100$$

where A_s_ is the absorbance of the strains, A_b_ is the absorbance without H_2_O_2_, and A_c_ is the absorbance of the control.

The SA scavenging ability of our potential probiotic strain was measured by following the method of Sui and Liu [1] and calculated according to Formula (8). The absorbance was measured at 329 nm.

(8) $$\mathrm{SASA}\;\%=\left[\left(A_{\mathrm{control}}-A_{\mathrm{sample}}/A_{\mathrm{control}}\right)\right]\times 100$$

where A_control_ is the absorbance of the control reaction, and A_sample_ is the absorbance of the LAB culture.

The RHP survival ability of the culture was assessed using the method of Li and Zhao [32] with some minor modifications in the hydrogen peroxide concentrations (0.2, 0.4, 0.6, 0.8, 1.0, and 2.0 mmol L^−1^) (GHTECH, Shantou, China). The optical density (OD) was measured at 600 nm. The survival ability of the strains was calculated according to Equation (9).

(9) $$\mathrm{SADPPH}\;\%=\left[1-\left(A_{\mathrm{sample}}-A_{\mathrm{blank}}/A_{\mathrm{control}}\right)\right]\times 100$$

### 2.5. Short-Chain Fatty Acids (SCFAs) Analysis

The levels of short-chain fatty acids (acetic acid, propionic acid, butyric acid, isobutyric acid, and valeric acid) in the fermented broth and mice feces were measured by using gas chromatography (GC-2014C Shimadzu corporation, Kyoto, Japan). The methodology introduced by Kang and Kim [16], and Zhang and Fan [34] was used to extract and measure SCFAs with minor modifications. In brief, ~0.2 g of feces and 0.8 mL of supernatant of the fermented culture were mixed with 1.0 mL of ether and 50% hydrochloric acid (H_2_SO_4_) for acidification. The mixture was centrifuged at 10,000× *g* for 15 min to collect the organic phase. The samples were subjected to GC which was equipped with a DB-FFAP separation column (30 m × 0.25 µm × 0.25 μm, Agilent Technologies, Palo Alto, CA, USA).

### 2.6. Animal Experiment

Twenty 6–8-week-old BALB/c white mice were purchased from Xi’an Jiaotong University health science center (China). The mice were maintained in a bio-security isolation unit under a controlled environment with free feed and water access (2 mice in one cage). After acclimatizing for two weeks, mice were divided into two experimental groups (n = 10), namely the control group and administration group. The challenge group was orally administered 200 µL of 10^8^ fresh culture daily, while the control group was gavaged with 200 µL of PBS for the remaining two weeks. After the 4th week, mice were euthanized by injection with 10% chloral hydrate. The experiment was performed per the guidelines of the National Institutes of Health and was approved by the Animal Protection and Use Committee of Northwest Polytechnic University (license number: SCXXK 2017003). The weight of the kidney, spleen and liver was measured to calculate the body weight index. For histopathological evaluation, colonic tissues (~1 cm) were fixed overnight in 4% paraformaldehyde (Sinopharm, Shanghai, China), subsequently embedded in paraffin and sliced into sections of 5 μm thickness for hematoxylin and eosin analysis (H&E). An Olympus microscope (Olympus Corporation, Shinjuku, Tokyo, Japan) was used to observe and photograph the stained slides [35].

### 2.7. The 16S rRNA Gene Sequencing of Gut Microbiota in BALB/c Mice

In this study, the method introduced by Lee, Kim [17] was used to analyze the BALB/c mouse gut microbiota. Briefly, the total bacterial DNA was extracted from about 200 mg of cecum content using the DNA isolation kit of PowerFecal QIAamp 96 QIAcube HT (QIAGEN kit, Shanghai, China). The samples were collected from five mice after fourteen days of administration. The purity and concentration of extracted DNA were verified by Nano-Drop (DeNOVIX-DS-11 Spectrophotometer, Wilmington, NC, USA) and agarose gel (Hydra Gene. Co., Ltd., Xiamen, China). Then, the genomic DNA was amplified as a template for hypervariable V3-V4 regions of 16S rRNA genes. The PCR reaction was conducted under the same conditions described in the cited method with a final extension phase at 72 °C for 5 min. The mixture contained: DNA template at a concentration of 50 ng (2 µL), Gflex PCR buffer (15 µL), primers 343F-798R (2 µL), and Tks Gflex DNA polymerase (0.6 µL). The PCR products were visualized after gel electrophoresis and purified by AMPure XP beads. Afterwards, the final amplicon was quantified using a Qubit dsDNA assay kit and sequenced using the IlluminaMiSeq system at BEIJING BIOMARKER (Beijing, China).

### 2.8. Bioinformatic Analysis

Raw sequences data were converted to FASTQ format and bioinformatically analyzed using the BMK Cloud platform (http://www.biocloud.net/. accessed on 15 July 2022). The low-quality end-paired sequences with an average score < 20 were detected by Trimmomatic software to cut off the ambiguous bases of chimera readings. After trimming, the high-quality reads with 75% of bases above Q20 were assembled using FLASH software [36]. In contrast, all of the effective readings were clustered to generate operational taxonomic units (OTUs) using UPARSE software (Ver. 7.1) with a similarity of 97%. Then, the representative bacterial OTUs were annotated on SILVA database, ver. 123, and blasted against the ribosomal database project classifier (RDP, ver. 2.2). Amplicon sequence variants analysis (OTU/ASV) represented the real biological sequences to distinguish the differences between sequences in the precision of single nucleotides and the identification of strain at the species level. The gut microbiota diversity was evaluated as follows: (1) species annotation and taxonomic analysis were conducted by using SILVA as a reference database. (2) Alpha diversity analysis reflected the richness and diversity of individual samples (within groups), and had multiple metrics: Chao1, Ace, Shannon, Simpson, Coverage, and PD-whole-tree indexes. (3) QIIME software was used to analyze beta diversity to compare the degree of similarity between species (between the groups). It mainly used four algorithms, such as binary jaccard, bray curtis, weighted unifrac (limited bacteria), and unweighted unifrac (limited bacteria). We measured the principal component analysis (PCA) and (PCoA) to reflect differences in multiple sets of data on a two-dimensional coordinate plot and the unweighted pair-group method with arithmetic mean (UPGMA) cluster tree, which is an analysis method that combines cluster trees with abundance column charts. (4) Significance analysis of difference between groups was estimated by means analysis of variance (ANOVA), which is also known as the F test, and through an evolutionary branching diagram of LEfSe analysis which combined nonparametric Kruskal–Wallis and Wilcoxon rank sum tests with linear discriminant analysis (LDA). (5) Correlation and association analysis was performed to discover the abundance of and changes in each species in each sample. Based on this analysis, the coexistence relationship and interaction between species in environmental samples, the important model information, and the formation mechanism of phenotypic differences between samples can be explained. (6) Functional gene predictive analyses such as Phylogenetic Investigation of Communities by Reconstruction of Unobserved States (PICRUSt2) software were used to predict the functional characteristics based on the abundance of marker gene sequences in samples; this can also predict the pathway of the entire community by combining the KEGG pathway information of genes. In addition, Clusters of Orthologous Groups of proteins (COG) is a prokaryotic homologous protein cluster database commonly used as protein function classification database for prokaryotes. Ecological Function Prediction (FAPROTAX) is a tool more suitable for predicting the ecological functions of environmental samples and for bacteria identification at the genus and specie levels. We performed all of the analysis by using BMKCloud platform tools.

### 2.9. Statistical Analysis

In the present study, all data were analyzed in triplicate with Statistic 5.5 software. One-way ANOVA and Student’s *t*-test were performed to examine significant differences between various groups. Values of *p* < 0.05 were considered statistically significant. Experimental data were presented as the mean ± standard deviation of the mean. Evolutionary analysis was performed by means of MEGA11. The Spearman’s correlation matrix was produced by GraphPad prism 8.0.

## 3. Results

### 3.1. Molecular Identification of Lactiplantibacillus plantarum NWAFU-BIO-BS29

The potential probiotic strain was isolated from Chinese traditional fermented milk. The 16S rRNA nucleotide sequence has been deposited in NCBI database under GenBank accession number ON340624. See Table S1 for the sequence and Figure S1A for evolutionary analysis of the maximum likelihood method and Hasegawa–Kishino–Yano model.

### 3.2. Probiotics Properties

#### 3.2.1. Antibacterial Activity and Acid and Bile Salts Tolerance

**Figure 1:**
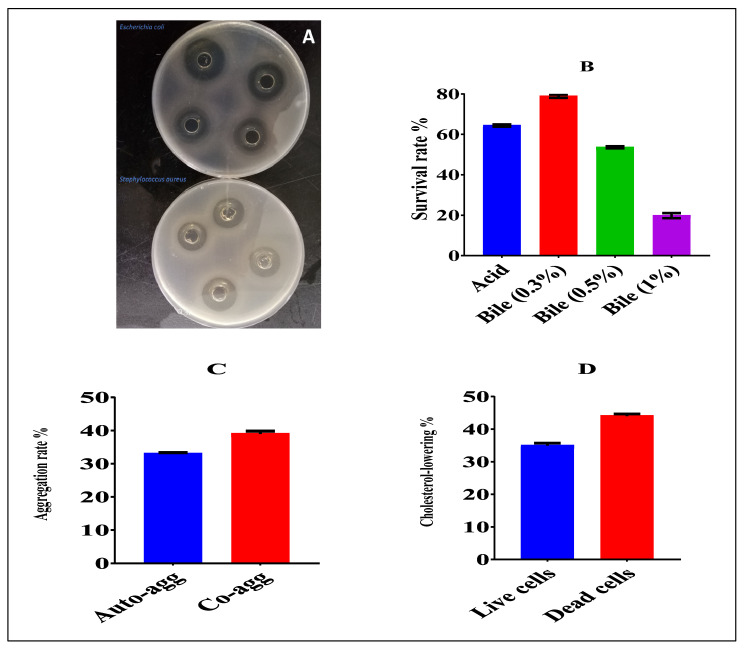
(**A**) Antibacterial activity of *Lactiplantibacillus plantarum* NWAFU-BIO-BS29 against Gram + and − pathogens; (**B**) acid and bile tolerance; (**C**) aggregation; (**D**) cholesterol-lowering activity.

The antibacterial activity of CFS against indicator strains of *Staphylococcus aureus* ATCC 25923 and *Escherichia coli* ATCC25922 showed large inhibition zones of 25 and 21 mm, respectively—see Figure 1A.

As shown in Figure 1B, the acid tolerance was 64.4%. Likewise, the bile tolerance ranged between 78.92%, 53.61%, and 19.83% in the presence of 0.3, 0.5, and 1% of bile salt, respectively.

#### 3.2.2. Aggregation and Cholesterol-Lowering Abilities

A high increase in auto-aggregation activity was observed at 12 h with an average value of 33.01 %, while the co-aggregation at 4 h had an average value of 38.96%—see Figure 1C.

The in vitro cholesterol-lowering ability indicated that this strain remarkably reduced cholesterol levels by more than 34.95% in live cells and 44.02 in dead cells—see Figure 1D.

### 3.3. In Vitro Safety Evaluation

Safety evaluation is the most important feature of feed additives or functional food. According to the EFSA’s guidance regarding standard antibiotic susceptibility cut-off values [37], this strain had high sensibility against the tested antibiotics—see Table S2. Assessment of hemolytic activity did not display any zones around the colonies on the blood plates (g–haemolytic). At the same time, the presence of 16 VFs in the genomic DNA of *Lactiplantibacillus plantarum* NWAFU-BIO-BS29 was not detected—see Table S3.

### 3.4. Antioxidant Activity Analysis

**Figure 2:**
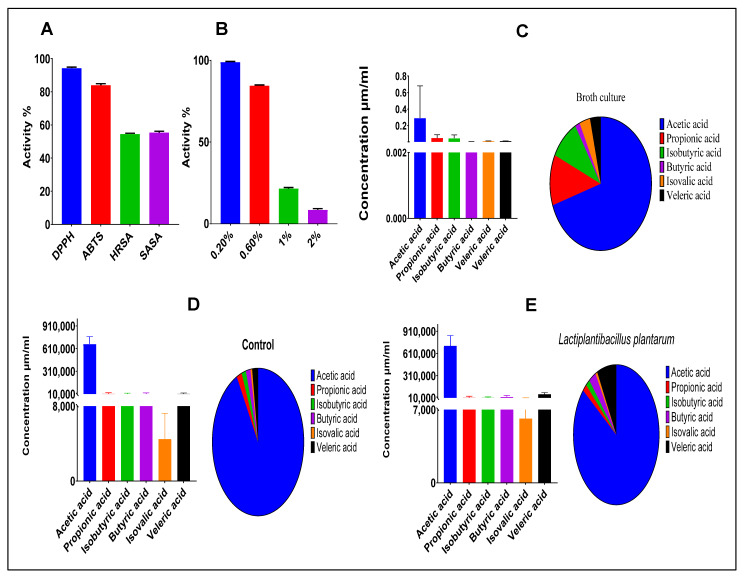
(**A**) Antioxidant activities. (**B**) Resistance to hydrogen peroxide. (**C**) SCFA profile of *L. plantarum* NWAFU-BIO-BS29 culture. (**D**) SCFA profile of feces of the un-administrated group. (**E**) SCFA profile of feces of the administrated group.

The antioxidant activities of our strain exhibited various radical scavenging capacities. The ABTS assay showed a scavenging activity of 83.99 %. Likewise, the DPPH assay of the CFS had a scavenging activity of 94.30 %. Furthermore, hydroxyl radical scavenging activity showed high activity, reaching 54.61%. Meanwhile, superoxide anion scavenging activity had a value of 55.45%—see Figure 2A. Finally, the tested strain had a high ability to grow in different concentrations of hydrogen peroxide (0.2, 0.6, 1.0, and 2.0 mM). The average growth rates were 95.29, 84.38, 14.98, and 7.72%, respectively—see Figure 2B.

### 3.5. SCFAs Present in Culture Medium and Feces

The total SCFAs produced during the fermentation of carbohydrates was 0.418 μm/mL. For comparison, the most abundant is acetic acid, at a concentration of 0.291 μm/mL, followed by propionic acid (0.051 μm/mL)—see Figure 2C. The SCFA profile of the feces samples showed that the administered and control groups had concentrations of 832,044 and 727,912 μm/mL, respectively—see Figure 2D,E.

### 3.6. Effects of L. plantarum NWAFU-BIO-BS29 on Body Weight, Organs Index, and Colon H&E in BALB/c Mice

**Figure 3:**
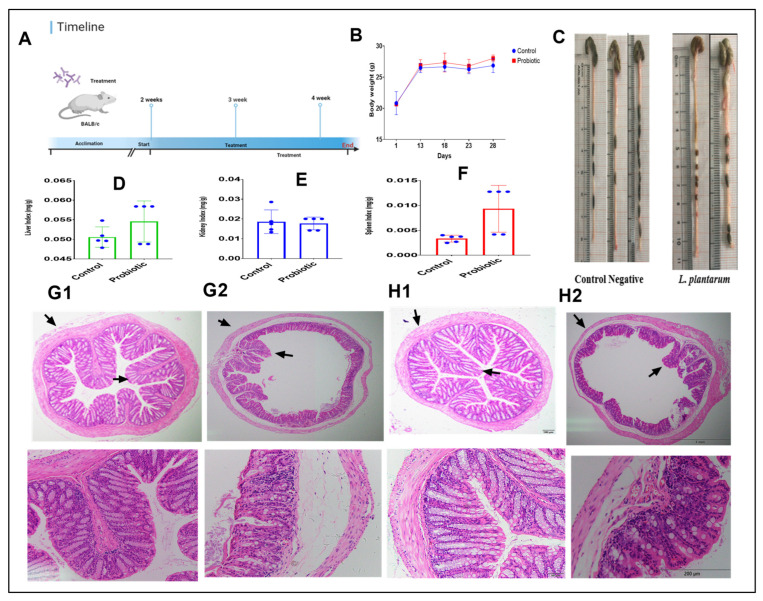
(**A**): Experimental schedule. (**B**) Body weight. (**C**) Intestinal length. (**D**) Liver index. (**E**) Kidney index. (**F**) Spleen index. (**G1**) Colon of the control group. (**G2**) Cecum of the control group. (**H1**) Colon of the administered group. (**H2**) Cecum of the administered group.

Figure 3A shows the timeline of the animal experiment. Accordingly, there was no significant change in the body weights of the challenge group and the normal group after 4 weeks (Figure 3B). In contrast, treatment with 8 × 10^9^ CFU/day of *L. plantarum* improved the intestinal length (Figure 3C) and the average value of the liver index (Figure 3D) and spleen index (Figure 3F), while the kidney index indicated there was no difference among them (Figure 3E). H&E analysis showed that the administrated group has healthier villi and a thicker intestinal prier of the colon and cecum (Figure 3H1,H2) compared with the control group (Figure 3G1,G2).

### 3.7. Effect of L. plantarum NWAFU-BIO-BS29 on the Gut Microbiota in BALB/c Mice

**Figure 4:**
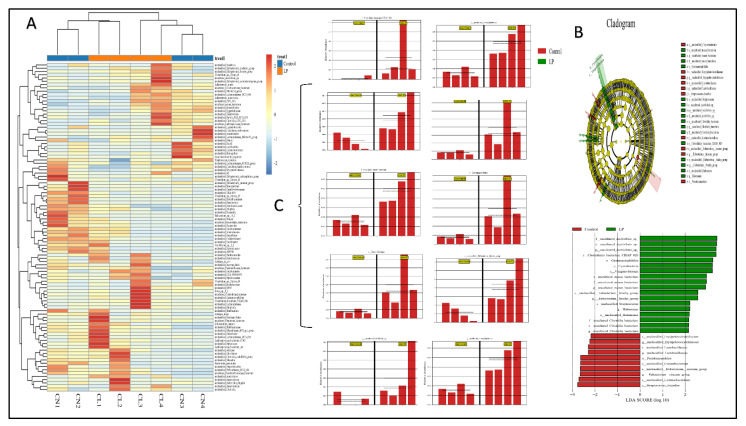
(**A**) Heat map of distribution of sample communities. (**B**) LEfSe analysis. (**C**) Bar of significant differences between groups.

**Figure 5:**
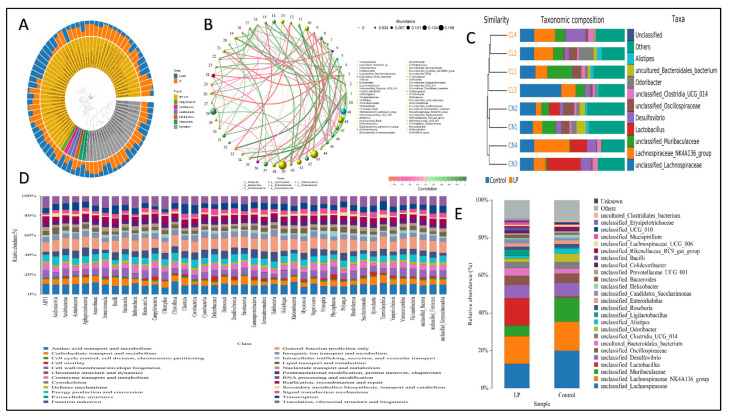
(**A**) Species evolutionary trees. (**B**) Diagram of species at the genus level. (**C**) UPGMA cluster tree. (**D**) Histogram of COG metabolic pathway. (**E**) Species abundance cluster heat map.

In each experimental group, we analyzed the gut microbiota diversity of fecal samples to examine the effects of *L. plantarum* NWAFU-BIO-BS29 administration. The quality of data sequencing was assessed by processing the number of sample sequences and their length, as shown in Table S4. For OTU/ASV analysis, see Table S5, and for the real species in samples, see Figure S2A. Moreover, Figure 4A and Figure 5A,E show the species abundance cluster heat map, the distribution of sample communities of species evolutionary trees and the species distribution map, respectively. Furthermore, for alpha diversity analysis, see Figure S2B, and see Figure S3E–G for a boxplot of the differences between groups, the samples’ dilution curves, the samples’ Shannon Index curves, and the samples’ rank-abundance curves. Likewise, beta diversity analysis was performed to calculate the distance between samples in order to obtain the β value using the principal component analysis (PCA) (Figure S2C) and (PCoA) analysis plot (Figure S2D), as well as the unweighted pair-group method with arithmetic mean (UPGMA) cluster tree (Figure 5C) and the clustering heatmap to generate a phylogenetic species tree (Figure S2E). Moreover, significant differences between samples were tested by ANOVA analysis (Figure S3F) and LEfSe analysis (Figure 4B,C). Spearman rank correlation analysis was carried out, and the data with a correlation greater than 0.1 and a *p*-value of less than 0.05 were screened to construct a correlation network for the Node Zi-Pi distribution diagram (Figure S3D) and the diagram of species at the genus level (Figure 5B). PICRUSt2 was used to align the feature sequence (16S rRNA) with the reference sequence (aliign) of the microbial genome database (IMG) to construct an evolutionary tree, find the “nearest species” of the feature sequence, and predict the genetic information of unknown species present in the diagram of the KEGG metabolic pathway (Figure S3C). The Clusters of Orthologous Groups of proteins (COG) protein cluster database was used for predicting the protein function; the analysis results are shown in the figures below, where Figure 5D and Figure S3A depict histograms of the COG metabolic pathway and function classification chart and Figure S3B presents FAPROTAX for the prediction of biogeochemical cycle processes.

### 3.8. Correlation

**Figure 6:**
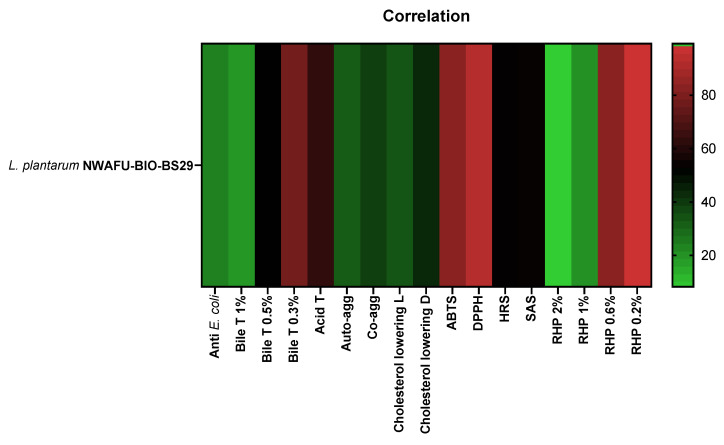
Spearman’s correlation matrix.

A Spearman’s correlation coefficient was used to evaluate the relationship between different parameters of probiotic properties (antibacterial and antioxidant activity, acid and bile salt tolerance, aggregation capability, and cholesterol-lowering ability). The matrix (Figure 6) shows the correlation relationship between them.

## 4. Discussion

This study investigated the probiotic properties of *Lactiplantibacillus plantarum* NWAFU-BIO-BS29 isolated from fermented milk samples and its interference with the gut microbiota in a mouse model. The most common in vitro methods used to detect the viability and activity of probiotics involve testing their tolerance to highly acidic pH (about 3.0) and bile salt conditions (about 0.3%) to simulate human gastric and intestinal fluids [5]. We found that the tested strain is acid- and bile salt-tolerant, with a survival rate of 83.26% at pH 3.0. Meanwhile, the strain’s ability to grow and metabolize at bile concentrations of 0.3%, 0.5% and 1% showed remarkable bile salt resistance, with a proportion of growth averaging 78.92%, 53.61% and 19.83%, respectively. This result is significantly better than that reported previously by Qian and Long [6]. They found that *Lactobacillus plantarum* YS2 (LP-YS2) has a growth rate of 68.05% at pH 3.0 and 19.85%, 15.0%, and 8.35% at percentages of bile salt of 0.3%, 0.5%, and 1.0%, respectively. In contrast, strong antibacterial activity against pathogens was observed, such as *Escherichia coli* ATCC25922 and *Staphylococcus aureus* ATCC25923, which is higher than that reported by Azat and Liu [5] and Metrouh and Fares [38]. This activity could be explained due to its ability to produce antimicrobial substances such as lactic acid, acetic acid, diacetyl, fatty acids, aldehydes, and bacteriocins. In addition, the functional characteristics of its auto- and co-aggregation capabilities demonstrated that it had a high auto-aggregation ability of 34.4% to adhere to itself and a co-aggregation ability with entero-pathogens of up to 83.96%, which plays an important role in forming a barrier that prevents the colonization of harmful enteric pathogens. Fhoula and Rehaiem [24] reported that *Lactobacillus rhamnosus* R4 had co-aggregation ability of 45.83% after 24 h and co-aggregation ability of more than 30% after 1 h, compared to our study, where the auto and co-aggregation were evaluated after 14 and 4 h, respectively. In addition, the cholesterol-reduction ability of NWAFU-BIO-BS29 was assessed. The strain displayed a high level of cholesterol-lowering ability at rates of 34.95% in live cells and 44.02% in dead cells. Interestingly, the supernatant of the bacterial culture was able to reduce cholesterol to a greater extent compared to the intact cells; this might be a result of the activity of certain components in the supernatant, in addition to those existing in the live cells [5]. These findings are significantly higher than the reported ability of *L. rhamnosus*, which reduced cholesterol in vitro by 22% [25]. A WHO report of 2015 showed that a 10% cholesterol reduction in serum could decrease the incidence of heart disease by 50% in 40-year-old men [7].

The safety assessment of potential probiotic strains is the most important factor. Screening for the presence of virulence factor genes showed that *Lactiplantibacillus plantarum* NWAFU-BIO-BS29 does not carry any VFs and can safely be used. This was consistent with numerous studies on *Lactobacilli* species such as *E. faecium* CM33 and *E. durans* LAB18s, which were found to be negative for all tested VFs of *vanA*, *vanB* and *vanC2* and other VFs of collagen adhesion (*acm*), aggregation substance (*agg* and *asa*), cytolysin (*cylA*), gelatinase (*gelE*), aggregation adhesion collagen protein (*ace*), *bopB* (beta-phosphoglucomutase), *bopC* (aldose 1-epimerase), *bopA* (putative glycosyltransferase), and *bopD* (sugar-binding transcriptional regulator) and resistance genes of *vanA*, *vanC1* and *vanC2/3* [12,28]. On the other hand, some studies reported *L. reuteri* VB4, *S. salivarius* NBRC13956, and *Lb. plantarum* ST8Sh to have VFs such as *asa1*, *esp*, *efaA*, and *ace* and resistance genes of *vanC 2/3* and *vanC1* [4,12,31]. Meanwhile, *tet(M)* was detected in *L. plantarum*, *L. curvatus*, *Lactobacillus casei, L. acidophilus*, *L. gasseri* and *L. crispatus*, the *tet(W)* gene was detected in strains of *L. crispatus*, *L. johnsonii*, and *L. reuteri*, while the *erm(B)* erythromycin resistance gene was detected in *L. reuteri*, *L. fermentum*, *L. casei*, *L. plantarum*, *L. acidophilus*, *L. gasseri*, *L. rhamnosus* and *L. johnsonii* [27]. The presence of VFs in the strain’s genomic DNA may lead to the transfer of these genes to the host or pathogenic bacteria, particularly the vancomycin resistance gene, which is the last antibiotic that works effectively against multidrug-resistant pathogens [39]. According to the EFSA, safe strains should not have any virulence factors and should not exceed the cut-off values of acquired resistance [40]. Other safety aspects that need to be evaluated are hemolytic activity and antibiotic resistance. In this study, the results exhibited no hemolytic activity (g-hemolysis). The strain was highly susceptible to common antibiotics used in animal feed (kanamycin, gentamycin, ampicillin, tetracycline, streptomycin, vancomycin, erythromycin, and chloramphenicol). A study by Klare and Konstabel [27] tested 12 LAB species against 13 antibiotics; they found that three *Lactobacillus* strains were highly resistant to one or more of streptomycin, erythromycin, clindamycin, and oxytetracycline. As stated by the FEEDAP-panel, resistant bacteria to antibiotics of human and veterinary importance should not be used as food and feed additives [27]. However, our strain does not have a potential risk for horizontal gene transfer. Since healthy people take in probiotics as food supplements, a healthy mouse model was used for the study with the commonly used amount of probiotics in vitro and in vivo without determining the concentration that produces the maximum effect [17]. During the feeding with NWAFU-BIO-BS29, there was no effect of oral administration on the body weight or mortality, and also no abnormal changes in their eyes, skin, or mucous membranes; no abnormalities in body movement, behavior, or gait; and no tremors, convulsions, drooling diarrhea, sleepiness, or other symptoms. In a preceding study, the feeding of 10^10^ CFU/mL of LAB isolates did not trigger an inflammatory response or influence body weight or show general signs of disease [41]. The intestine is the first organ to be exposed to the test substance, whereas the spleen, kidneys, and liver are vital immune organs responsible for metabolism [42]. To explore histopathological damage to the organs, we dissected the mice and harvested these organs to calculate the organ indexes, which demonstrated that there was remarkable improvement in the administered group. Intestinal organs (colon and cecum) were selected for HE staining to examine pathological changes. All abnormalities or histopathological changes were absent in the tissue’s structural makeup. The intestinal mucosa had a normal shape and structure as a result of the absence of goblet cell loss; the crypt was shallow and clear, and without epithelium necrosis or detachment from lamina propria, and the intestinal villi were neatly arranged as in the untreated group (Figure 3G1–H2).

The antioxidant activity was evaluated by means of chemical assays which exhibited various degrees of potent scavenging activities—83.99% for ABTS, 94.30% for DPPH, 54.61% for hydroxyl radical scavenging activity, 55.45% for superoxide anion scavenging activity, and 95.29%, 84.38%, 14.98%, and 7.72% for resistance to hydrogen peroxide at concentrations of H_2_O_2_ of 0.2, 0.6, 1.0, and 2.0 mM, respectively. Previous studies noted less antioxidant activities of *L. plantarum* C88, *L. rhamnosus* R4, *E. hirae* K4-8, *L. plantarum* C88 and *L. rhamnosus* R4 which showed scavenging activity of (53.05%), (53.78%), (32.11%), (44.31%), and (45.79%), respectively [5,32]. Cell integrity is an important factor that affects scavenging activity; the intact cells of NWAFU-BIO-BS29 exhibited better behavior for hydroxyl radical scavenging activity than those of cell-free extracts. We inferred that it is probably due to LAB extracellular antioxidant components such as polysaccharides, peptidoglycan, and teichoic acid. Moreover, some intracellular enzymes such as NADH-oxidase, NADH-peroxidase, and superoxide dismutase (SOD) were likely obtained after breaking up the bacterial cells [5].

SCFAs production is critical in preventing and decreasing inflammation and could suppress the growth of pathogenic intestinal bacteria, modulate gut-microbiota, improve lipid metabolism and the human immune function, lower intestinal pH, and promote the bioavailability of minerals such as magnesium and calcium. For example, propionic acid has antimicrobial activity against *Escherichia coli* and *Salmonella* spp. and anti-cholesterolemic effects [16]. Butyrate acid has recently been shown to induce antioxidants to suppress hepatic oxidative stress in rats [34]. The SCFA profile of the administered group showed a significantly higher ability to produce butyric acid and veleric acid (22,909 and 60,949 μm/mL), respectively than the control group (11,676 and 15,113 μm/mL). Furthermore, the total concentration of SCFAs was increased from 727,912 to 832,044 μm/mL. This result supported the in vitro antimicrobial activity and suggested that NWAFU-BIO-BS29 can be used to promote health benefits to the host.

The gut microbiome plays an important role in maintaining host health, which could supply various nutrients, regulate energy balance, improve the production of SCFAs, modulate the immune response, and defend against pathogens [43], whereas dysbiosis or an imbalanced gut microbiota of the host reflects negative shifts in abundance, diversity, and the relative distribution of the gut microbiome composition. Moreover, it would increase intestinal permeability, which causes metabolic endotoxemia and low-grade chronic inflammation through a Toll-like receptor-mediated inflammatory pathway [44]. Animal experiments have reported that the gut microbiome diversity and its richness could be improved by the dietary intervention of some probiotics, which exert beneficial effects by modulating the gut microbiome and their metabolites [45]. In this study, the dietary treatment by NWAFU-BIO-BS29 led to the regulation of the gut microbiota of mice, which increased the abundance of Firmicutes, Patescibacteria, Campylobacterota, Deferribacterota, Proteobacteria, and Cyanobacteria phyla (see Figure 5E) and decreased the abundance of *Bacteroides* and *Desulfovibrio.* While the species distribution histogram at the genus and species level showed an abundance of Lactobacillus, this explains the rise in SCFA levels in vitro and in vivo, which was confirmed by the functional gene predictive analysis of FAPROTAX function forecasting. The same findings have been reported by Lee and Kim [17] and Stojanov and Berlec [46]. Additionally, in this context, LEFSe exhibited a significant increase in the administered group at the genus level of: (*Vampirivibrionia*, *Acetivibrio* sp., *Clostridia bacterium*, *rumen bacterium*, *Eubacterium brachy group*, *Halomonas*, *Lactobacillaceae*, *Gastranaerophilales*, *Cyanobacteria, Clostridiales bacterium CIEAF 020*, *Lachnoclostridium*, and *Streptococcus*). Meanwhile, alpha diversity indicated that the administered group has richness in species due to the decreased in Inverse Simpson, Chao1, and Shannon indices (Figure S2B). A previous study by Cui and Guo [47] presented results consistent with these findings. Therefore, targeting changes in the gut microbiome using probiotics has emerged as a new approach with potential therapeutic intervention applications.

Accordingly, these results indicate that *Lactiplantibacillus plantarum* NWAFU-BIO-BS29 had cholesterol-lowering capability, was tolerant to gastric acidic and bile salts conditions, had antibacterial activity against pathogens, and additionally has a remarkable aggregation ability to prevent the adhesion of pathogens. The safety investigation revealed that it is a safe strain and considered a good probiotic due to its ability to raise the SCFAs and modulate the gut-microbiota. Therefore, these underlying mechanisms of NWAFU-BIO-BS29 can be proposed to increase the abundance of beneficial bacteria in the gut of mice and result in a decreased inflammatory response.

## 5. Conclusions

Nowadays, consumers are becoming more aware of healthy and natural foods. This work aimed to characterize the features of *Lactiplantibacillus plantarum* NWAFU-BIO-BS29 isolated from Gansu traditional fermented milk, considered a functional food containing beneficial probiotics. We have proven that this strain is safe to be supplemented in food and feed with no detection of VFs. In addition, it showed a high capacity to survive on simulated human gastric fluid conditions, and its supernatant is able to inhibit Gram + and − pathogens, and thus may have potential applications in food preservation. Meanwhile, the auto- and co-aggregation abilities indicated that adherence properties could play essential roles in the gut flora construction and formation of a barrier that prevents the colonization of harmful bacteria. Other important features include its ability to lower cholesterol in vitro and its great antioxidant activities. As can be seen from the SCFA profile and the 16S rRNA gene sequencing of the gut microbiota, NWAFU-BIO-BS29 has a promising effect on promoting health benefits in the host. In conclusion, *Lactiplantibacillus plantarum* NWAFU-BIO-BS29 exhibited high potential to be used as a probiotic strain that can be safely applied in functional foods and has a promising property to produce bio-preservative substances. This strain needs to undergo other in vitro and in vivo studies, especially in humans, to confirm the safety and health benefits. Moreover, the concentrations of probiotics that produce synergetic effects should be tested for identifying the maximum effect in future.

## Data Availability

The datasets generated for this study are available on request to the corresponding author.

## Supplementary Materials

The following supporting information can be downloaded at: https://www.mdpi.com/article/10.3390/foods11233766/s1, Figure S1. A; Neighbour-joining of the Phylogenetic tree based on 16S rRNA gene sequence. B; Haemolysis activity. C; Detection of the presence of 16 genes related to virulence, biogenic amines, and antibiotic resistance. Figure S2. A; the real species in samples. B; Boxplot of the difference between groups. C; PCA. D; (PCoA) analysis plot. E; heatmap phylogenetic species tree. F; ANOVA analysis. Figure S3. A; function classification chart. B; FAPROTAX. C; diagram of KEGG metabolic pathway. D; Node Zi-Pi distribution diagram. E; samples dilution curve. F; samples Shannon Index curve. G; samples rank-abundance curve. Table S1. Primers sequences of virulence genes were used to detect by PCR. Table S2. Antibiotic classification, MIC (mg/mL) profiles to inhibit LAB strains, and susceptibility type. Table S3. Detection of 16 (VFs) genes related to virulence, biogenic amines, and antibiotic resistance. Table S4. Statistics of sample sequencing data processing results. Table S5. Distribution table of the number of characteristics of each sample.
