# Supplementary material for: Probiotic of Lactiplantibacillus plantarum NWAFU-BIO-BS29 Isolated from Chinese Traditional Fermented Milk and Its Potential Therapeutic Applications Based on Gut Microbiota Regulation
Source: Foods. 2022 Nov 23;11(23):3766. doi: 10.3390/foods11233766 (PMC9738876; doi:10.3390/foods11233766)
Supplement: Supplementary file 1 [file foods-11-03766-s001.zip › foods-2016983-supplementary.pdf]

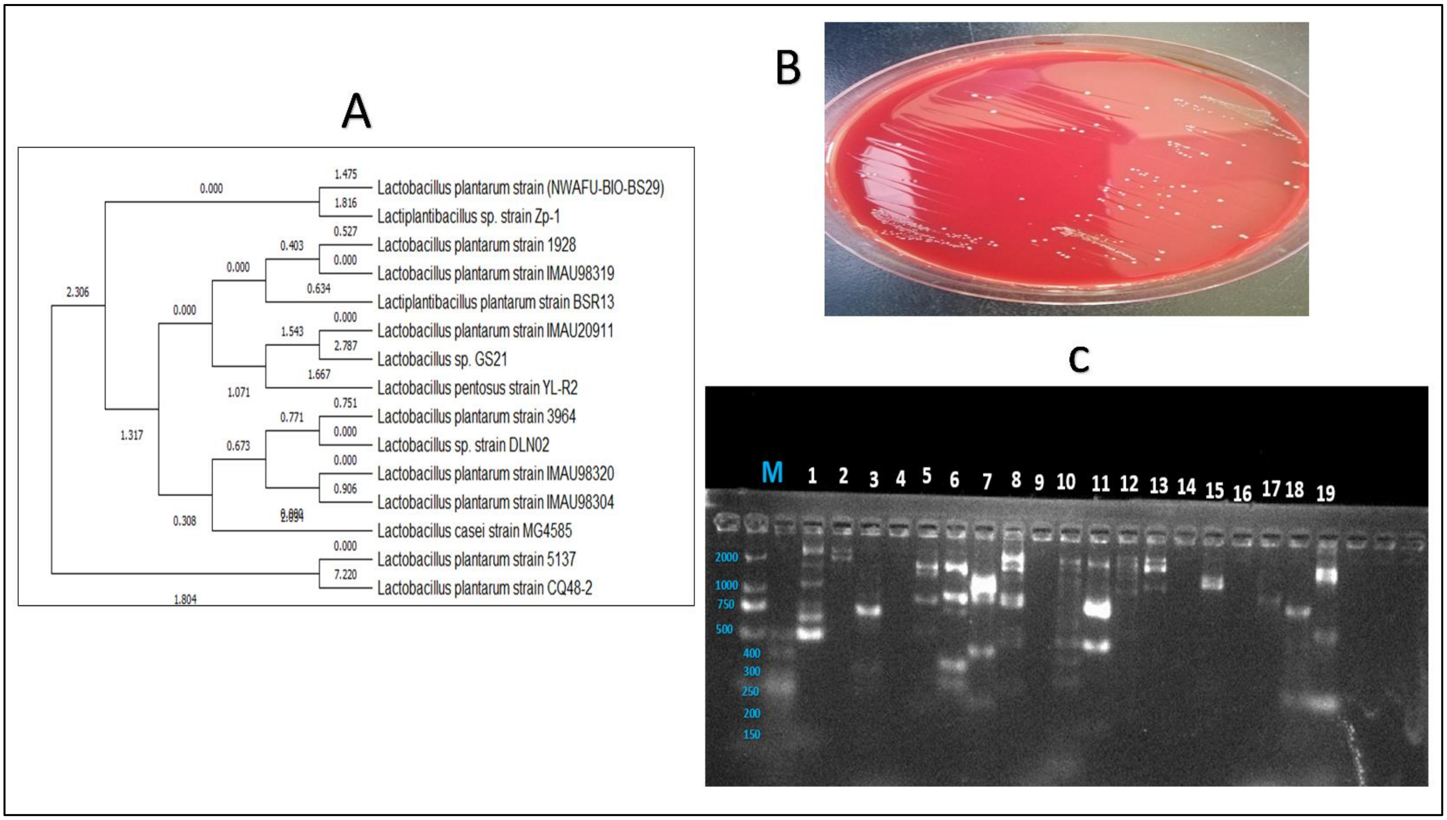

Figure S1. A. Neighbour-joining of the Phylogenetic tree based on 16S rRNA gene sequence. **B.** Haemolysis activity. **C**. Detection of the presence of 16 genes related to virulence, biogenic amines, and antibiotic resistance.

Note: Evolutionary analysis was conducted in MEGA11by Maximum Likelihood method. The evolutionary history was inferred by the Maximum Likelihood method and Hasegawa-Kishino-Yano model. The tree with the highest log likelihood (-28887.05) is shown. Initial tree for the heuristic search were obtained automatically by applying Neighbor-Join and BioNJ algorithms to a matrix of pairwise distances estimated using the Maximum Composite Likelihood (MCL) approach and then select the topology with superior log likelihood value. (www.megasoftware.net).

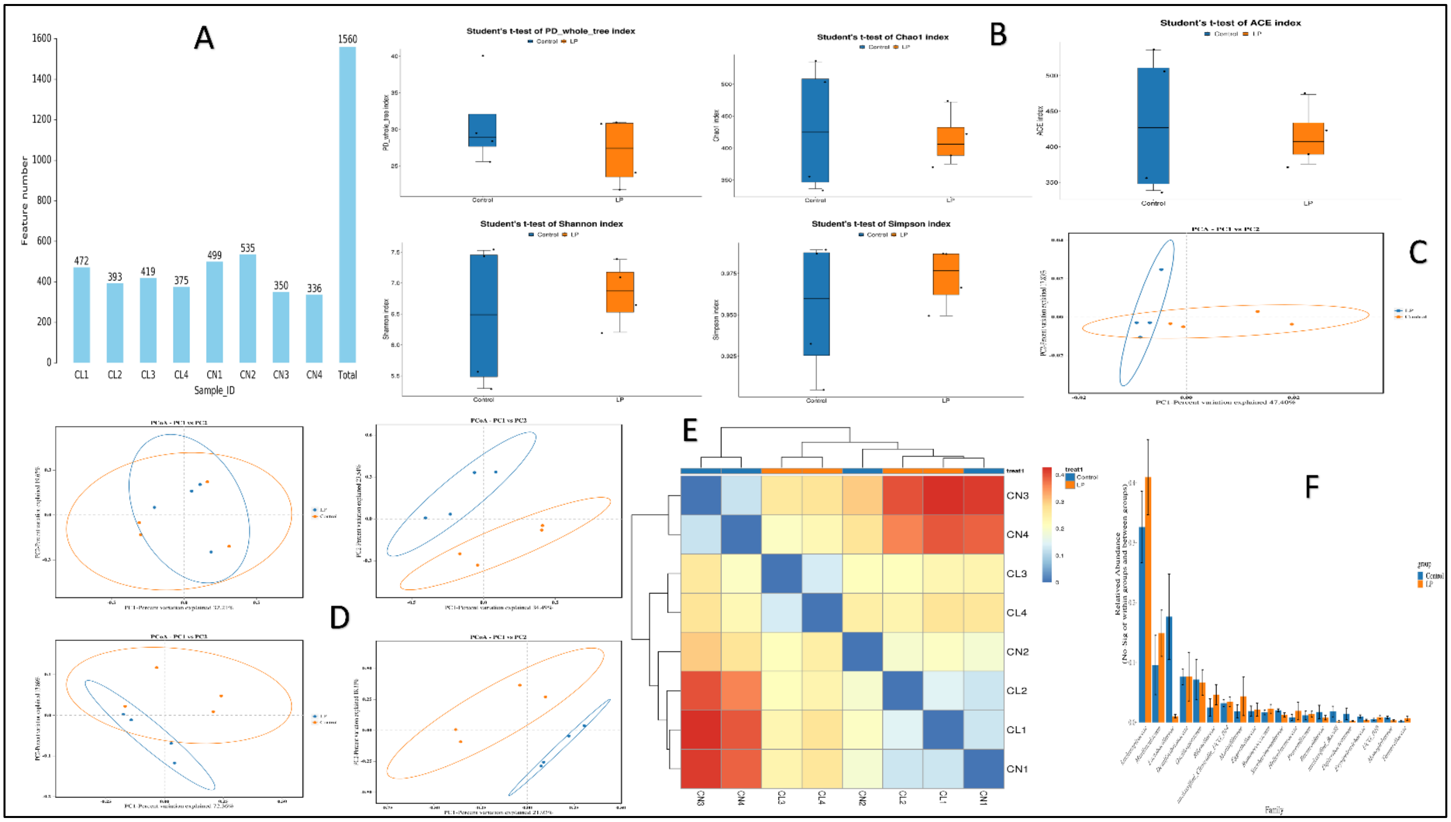

Figure S2. **A**; the real species in samples. **B**; Boxplot of the difference between groups. **C**; PCA. **D**; (PCoA) analysis plot. **E**; heatmap phylogenetic species tree. **F**; ANOVA analysis.

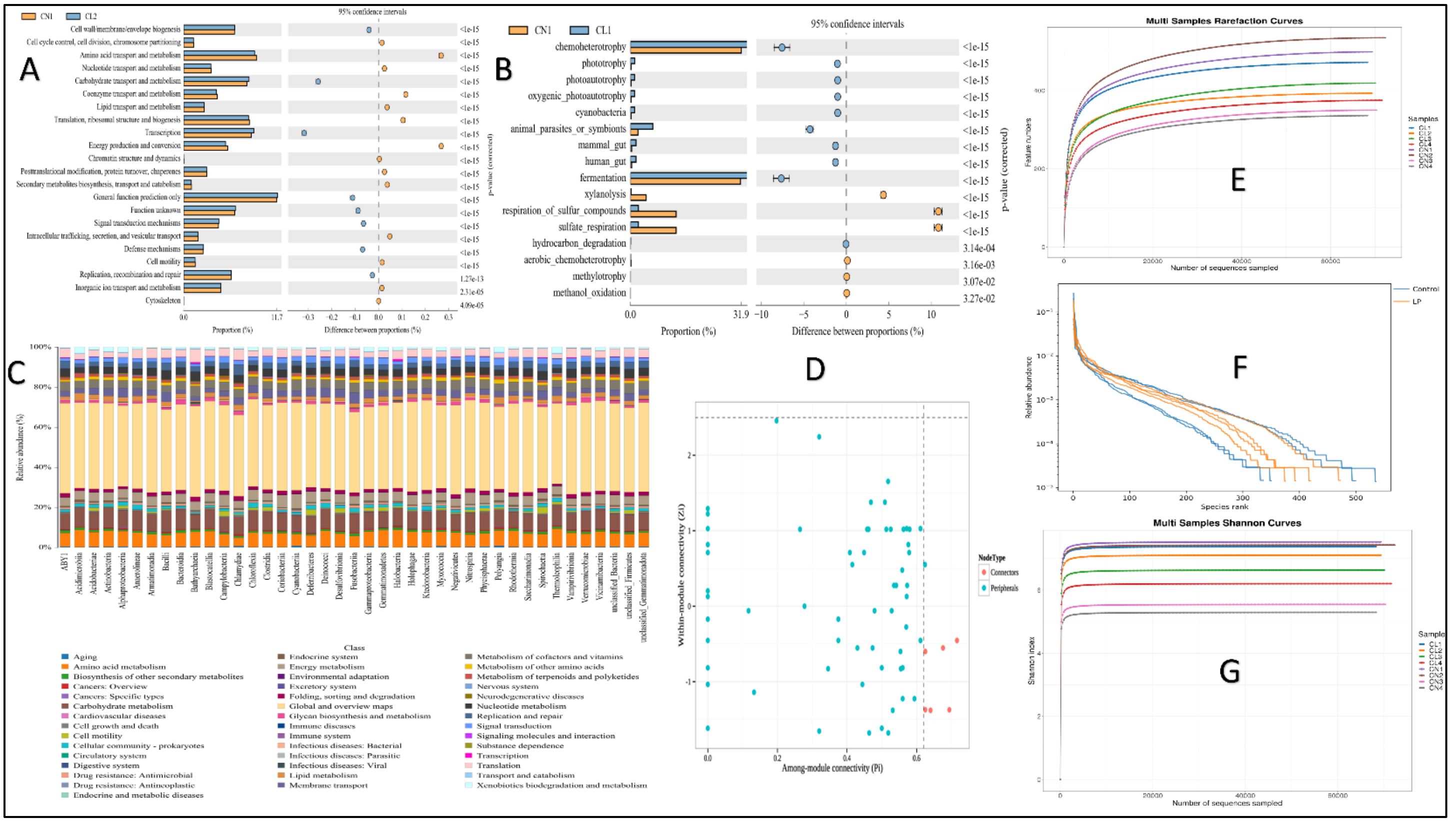

Figure S3. **A**; function classification chart. **B**; FAPROTAX. **C**; diagram of KEGG metabolic pathway. **D**; Node Zi-Pi distribution diagram. **E**; samples dilution curve. **F**; samples Shannon Index curve. **G**; samples rank-abundance curve.

Table S1. Primers sequences of virulence genes were used to detect by PCR.

No	Target gene	Primers
1	*fsrA-F*	ATGAGTGAACAAATGGCTATTTA
	fsrA-R	CTAAGTAAGAAATAGTGCCTTGA
2	*asa1-F*	GCACGCTATTACGAACTATGA
	asa1-R	TAAGAAAGAACATCACCACGA
3	*efaA-F*	GCCAATTGGGACAGACCCTC
	efaA-R	CGCCTTCTGTTCCTTCTTTGGC
4	*cpd-F*	TGGTGGGTTATTTTTCAATTC
	cpd-R	TACGGCTCTGGCTTACTA
5	*hyl-F*	ACAGAAGAGCTGCAGGAAATG
	hyl-R	GACTGACGTCCAAGTTTCCAA
6	*mur-2ed-F*	AACAGCTTACTTGACTGGACGC
	mur-2ed-R	GTATTGGCGCTACTACCCGTATC
7	*Int-F*	GCGTGATTGTATCTCACT
	Int-R	GACGCTCCTGTTGCTTCT
8	*tdc-F*	GAYATNATNGGNATNGGNYTNGAYCARG
	tdc-R	CCRTARTCNGGNATAGCRAARTCNGTRTG
9	*odc-F*	GTNTTYAAYGCNGAYAARCANTAYTTYGT
	odc-R	ATNGARTTNAGTTCRCAYTTYTCNGG
10	*hdc1-F*	AGATGGTATTGTTTCTTATG
	hdc1-R	AGACCATACACCATAACCTT
11	*aac(6')-Ie-aph(2")-Ia- F*	CCAAGAGCAATAAGGGCATA
	aac(6')-Ie-aph(2")-Ia- R	CACTATCATAACCACTACCG
12	*vanC2-F*	CTCCTACGATTCTCTTG
	vanC2-R	CGAGCAAGACCTTTAAG
13	*ermA-F*	TCTAAAAAGCATGTAAAAGAA
	ermA-R	CTTCGATAGTTTATTAATATTAG
14	*tet(K)-F*	TTAGGTGAAGGGTTAGGTCC
	tet(K)-R	GCAAACTCATTCCAGAAGCA
15	*catA-F*	GGATATGAAATTTATCCCTC
	catA-R	CAATCATCTACCCTATGAAT
16	*vat(E)-F*	ACGTTACCCATCACTATG
	vat(E)-R	GCTCCGATAATGGCACCGAC
17	*ant(6)-Ia-F*	ACTGGCTTAATCAATTTGGG
	ant(6)-Ia-R	GCCTTTCCGCCACCTCACCG

Table S2: Antibiotic classification, MIC (mg/ml) profiles to inhibit LAB strains, and susceptibility type.

Antibiotic classification [3]	Antibiotic	Cut-off values (mg/L)	Susceptibility type
Aminoglycoside	Kanamycin	64	S
	Gentamycin	16	S
Beta-lactams	Ampicillin	2	S
Protein synthesis inhibitor	Tetracycline	32	S
Cyclic peptides	Streptomycin	n.r.	S
Glycopeptide	Vancomycin	n.r.	S
Macrolides	Erythromycin	1	S
Amphenicols	Chloramphenicol	8	S

Note: (n.r.) not required, (S) Sensitive, (R) resistant, (I) intermediate to the antibiotics tested.

3 Zheng, Z.-Y.; Cao, F.-W.; Wang, W.-J.; Yu, J.; Chen, C.; Chen, B.; Liu, J.-X.; Firrman, J.; Renye, J.; Ren, D.-X. Probiotic characteristics of Lactobacillus plantarum E680 and its effect on Hypercholesterolemic mice. *BMC Microbiol.* **2020**, *20*, 239. https://doi.org/10.1186/s12866-020-01922-4.

Table S3: Detection of 16 (VFs) genes related to virulence, biogenic amines, and antibiotic resistance.

	Genes	Result
1. **Virulence**		
gelatinase	*fsrA*	○
aggregation	*asa1*	○
endocarditis	*efaA*	○
chemotactic factors	*cpd*	○
hyaluronidase	*hyl*	○
Enterococcus factors (surface protein)	*mur-2ed*	○
2. **Biogenic amines**		
tyrosine	*tdc*	○
ornithine	*odc*	○
histidine	*hdc1*	○
3. **Antibiotic resistance**		
aminoglycosides gentamicin	*aac(6')-Ie-aph(2")-Ia*	○
glycopeptides vancomycin	*vanC2*	○
macrolides erythromycin	*ermA*	○
tetracyclines tetracycline	*tet(K)*	○
other chloramphenicol	*catA*	○
streptogramin	*vat(E)*	○
streptomycin	*ant(6)-Ia*	○

Note ○; Not detect, ■; Detect.

Table S4: Statistics of sample sequencing data processing results

Sample ID	Raw Reads	Clean Reads	Denoised Reads	Merged Reads	Non-chimeric Reads
CL1	80291	79965	79636	73572	68483
CL2	80074	79778	79507	75012	69456
CL3	80199	79907	79685	74127	70239
CL4	79964	79708	79557	75070	71651
CN1	80220	79901	79675	73943	69551
CN2	79690	79391	79007	74867	72606
CN3	80280	80013	79797	75941	70559
CN4	79919	79648	79499	75095	68382

Note: The length distribution plot counts the number of sequences in the corresponding length range in each sample after quality control filtering.

Table S5: Distribution table of the number of characteristics of each sample

BMK_ID	OTU_Num	Seqs_Num
CL1	472	68294
CL2	393	69301
CL3	419	70133
CL4	375	71555
CN1	499	69320
CN2	535	72429
CN3	350	70473
CN4	336	68313

Note: (1) BMK_ID: sample number. (2) OTU_Num: Number of Features (OTU/ASV). (3) Seqs_Num: Feature corresponds to the number of reads.
